# Reviving Vision: A Case Report of Prosthetic Eye Restoration

**DOI:** 10.7759/cureus.58811

**Published:** 2024-04-23

**Authors:** Dhanashree A Minase, Seema Sathe, Anjali Borle, Mithilesh M Dhamande, Tanvi Jaiswal

**Affiliations:** 1 Prosthodontics, Sharad Pawar Dental College, Datta Meghe Institute of Higher Education, Wardha, IND

**Keywords:** prosthetic eye, lens, vision, orbital, eye prosthesis

## Abstract

Prosthetic eye implantation is a significant intervention for individuals facing ocular trauma or congenital defects. We present the case of an eight-year-old boy who underwent prosthetic eye implantation following enucleation due to a severe injury. The patient had suffered from impaired vision and psychological distress due to the visible absence of his natural eye. The prosthetic eye not only restored his facial symmetry but also revitalized his self-esteem and confidence. This case report highlights the successful outcome of prosthetic eye implantation in pediatric patients and underscores the importance of addressing both physical and psychological aspects of ocular trauma in young individuals.

## Introduction

The loss or disappearance of a facial feature, particularly an eye, can result in serious psychological and physiological issues [1]. A congenital condition, painful blindness, irreversible trauma, cancer, or sympathetic ophthalmia are some causes of eye loss [2]. Evisceration, enucleation, or exenteration are the three surgical methods that may be used, depending on the extent of the involvement [3]. The majority of patients go through a lot of stress, mostly from adjusting to the loss of functional incapacity and social reactions to their face deformity. The patient's physical and psychological recovery, as well as increased social acceptance, depend on replacing the lost eye as soon as possible [4,5]. Facial asymmetries and anterior orbital region volume loss can be avoided with early treatment of an ophthalmic socket. One of the main goals of replacing an ocular prosthesis in an ophthalmic socket is to help the patient manage the challenging rehabilitation process more effectively. It takes a multidisciplinary team approach and management to give the patient precise and efficient follow-up treatment and rehabilitation. Thus, to deliver a successful ocular prosthesis [6], the joint efforts of the plastic surgeon, ophthalmologist, and maxillofacial prosthodontist are necessary. Ocular prostheses can be produced to order or off-the-shelf. When an implant is positioned in the orbit, a patient's prosthetic rehabilitation is significantly improved [7].

## Case presentation

**Figure 1:**
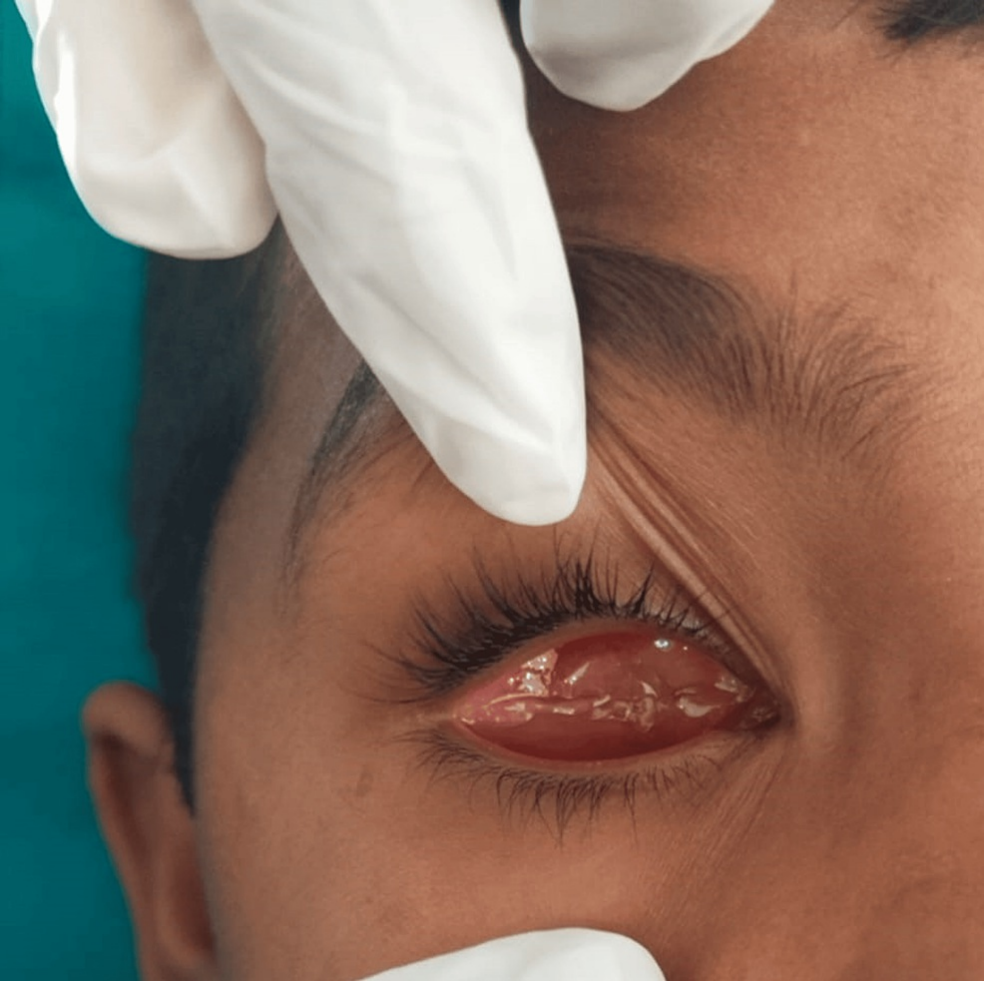
There are no signs of infection or pus discharge from the affected eye.

The main concern of an eight-year-old boy who presented to the prosthodontics department was that he was missing his right eyeball. The medical history indicated that the right eye had been traumatized and then enucleated. Upon examination of the eye socket, synchronous motions were observed, and the posterior wall of the anophthalmic socket was covered with a healthy conjunctiva that showed no symptoms of irritation or infection (Figure 1).

**Figure 2:**
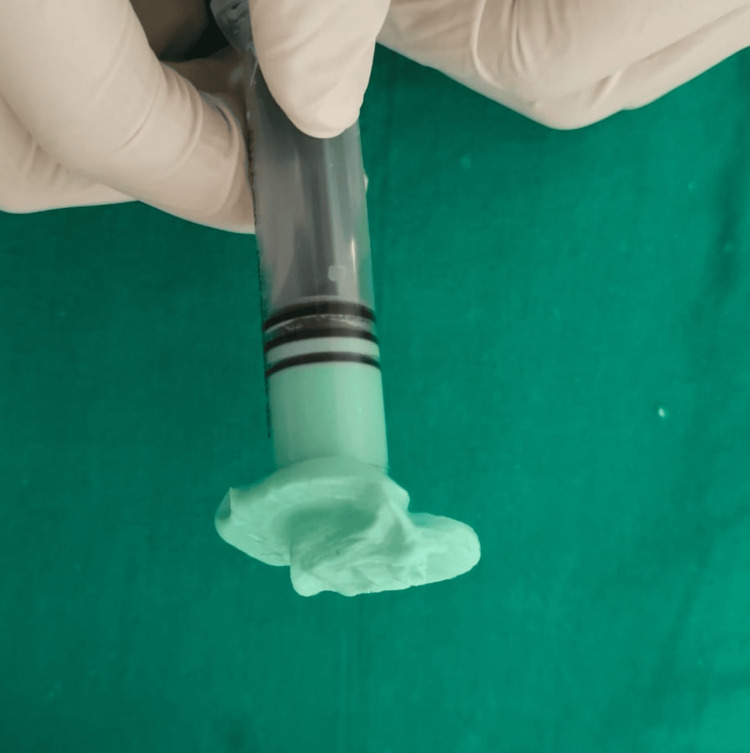
Impression made with irreversible hydrocolloid on a disposable syringe

With a custom-made sclera and a stock iris shell, a semi-customized ocular prosthesis was planned. After providing the patient and his parents with comprehensive information regarding the surgery, consent was obtained. The patient's eyelids were coated with petroleum jelly prior to taking the impression. Using a disposable syringe, alginate, the irreversible hydrocolloid substance, was used to create the initial impression. After mixing alginate to a fluid consistency and loading it into a disposable syringe, the patient was instructed to generate a functional impression by moving their eyes normally (Figure 2).

**Figure 3:**
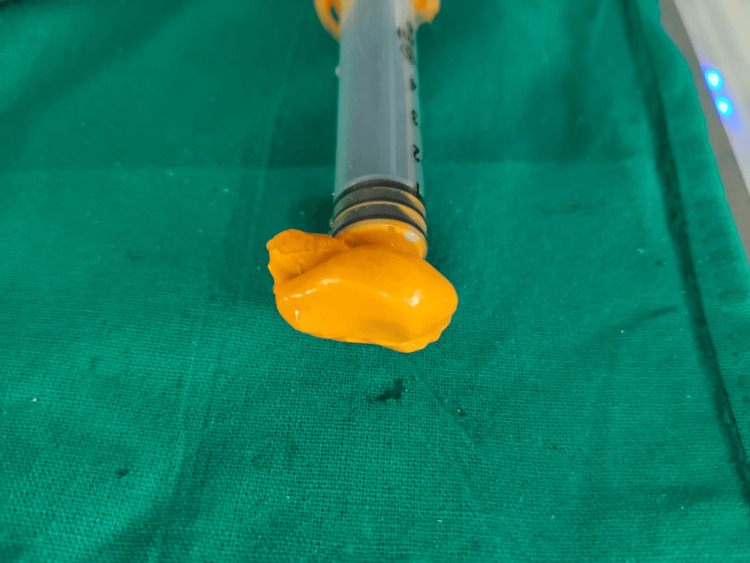
The final impression made with elastomeric material

The material was taken out and examined for bubbles once it had hardened. After that, it was filled to half the impression with a type II dental stone in a disposable glass. A second layer of dental stone was placed on top of the material, separating the media once it had set. Light body elastomeric material was used to create a final impression on a transparent acrylic custom-made shell (Figure 3).

**Figure 4:**
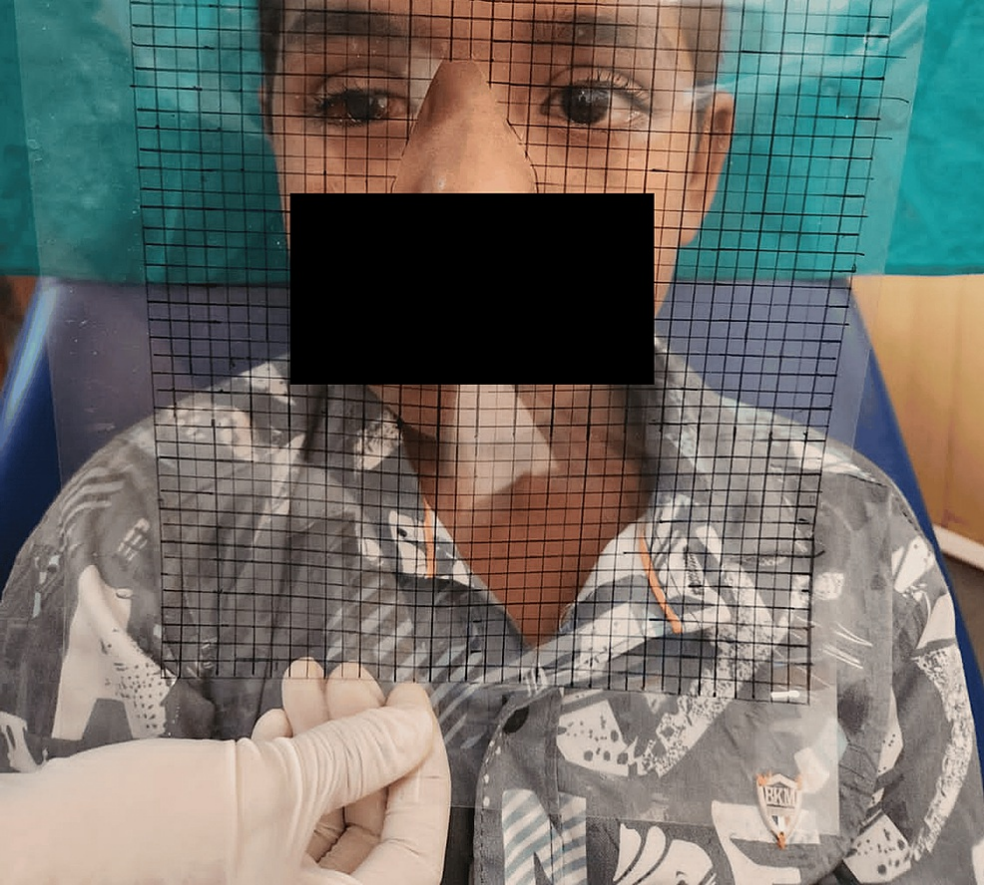
Iris positioning with the grid method

The main imprint and final impression were poured in the same way. After that, a mold was made using the finished cast, into which melted wax was poured. The result of it was the creation of wax patterns. Using the contralateral eye, which was then seated on the wax pattern with the use of a heated device, the size, color, and form of the iris were selected. The iris was positioned with the assistance of the grid method, and its location was verified using the same method (Figure 4).

**Figure 5:**
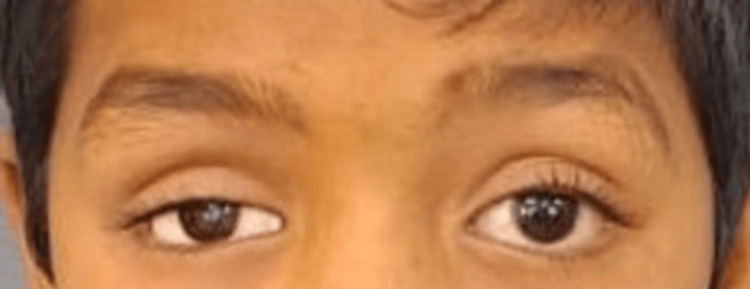
Final prosthesis insertion

The sclera's shade was noted in order to characterize it after fibers were injected. The patient was instructed to close their eyes, look up and down, and do side-to-side movements. Overextensions in the wax pattern were also removed. Transparent acrylic resin that had been heat-cured would then take the place of this wax. The manufacturer's recommendations were followed when employing the traditional curing method. The ocular prosthesis was completed and polished with caution to preserve its contour and convexity. The prosthesis was inserted into the socket after being cleaned and lubricated with an ocular lubricant to help with eye movements and preserve a tear film on the prosthesis (Figure 5).

Postoperative instructions were given to the patient for maintenance of hygiene and keeping the prosthesis in cold water at night. The patient was scheduled for follow-ups every three months. 

## Discussion

Through a surgical procedure known as evisceration, the entire intraocular contents are removed while the surrounding orbital adnexa, extraocular muscle attachments, and remaining scleral shells are preserved. An ocular prosthesis can be used to help a patient recover, at least aesthetically, following evisceration. The ocular prosthesis, built to order out of acrylic resin, makes close contact with the tissue bed [8]. Better contouring, color matching, and coordinated motions with the contralateral eye are some of the advantages of the customized ocular prosthesis over stock eyes [9]. The operator must invest more time and skill in customizing the iris [10, 11].

There are several ways to measure the iris's size and position, including using calipers, pupillometers, or visual assessment. Here, the iris was precisely located and positioned using a clear graph template, as opposed to relying solely on visual evaluation, which might result in inter-observer variability due to parallax errors and binocular vision problems. This approach is simple to use and can be implemented in any type of therapeutic setting, pupillometer was commonly used in older days [12, 13]. Each of these techniques had a subjective quality and was susceptible to operator bias. The "laser range finder" program was utilized in more recent methods by Belkhode et al. to measure the exact distances between the iris and the corners of the eyes using laser pointer equipment. [14]. Lanzara et al. located the iris on a digital image using Adobe Photoshop software [15]. The final prosthesis is made of acrylic resin, which is the most often used material due to its biocompatibility, aesthetic appeal, and longevity in terms of color, availability, and cost [13].

## Conclusions

The successful creation and fitting of a custom ocular prosthesis demonstrate the efficacy of tailored solutions in restoring the appearance of patients with ocular defects. This case highlights the importance of interdisciplinary collaboration and individualized care in achieving optimal outcomes. Moving forward, continued innovation and research in ocular prosthetics promise to further enhance the quality of life for affected individuals.
